# Independent Housing and Support for non-homeless individuals with severe mental illness: randomised controlled trial vs. observational study – study protocol

**DOI:** 10.1186/s12888-020-02712-y

**Published:** 2020-06-19

**Authors:** Christine Adamus, Sonja Mötteli, Matthias Jäger, Dirk Richter

**Affiliations:** 1 Center for Psychiatric Rehabilitation, Universitäre Psychiatrische Dienste Bern (UPD), Murtenstrasse 46, CH-3008 Bern, Switzerland; 2 University Hospital of Psychiatry and Psychotherapy, University of Bern, Bern, Switzerland; 3 Department of Psychiatry, Psychotherapy and Psychosomatics, University Hospital of Psychiatry Zurich, Zurich, Switzerland; 4 Psychiatrie Baselland, Liestal, Switzerland; 5 Departement of Health Professions, Bern University of Applied Sciences, Bern, Switzerland

**Keywords:** Residential rehabilitation, Supported housing, Non-homeless people, Serious mental illness, Randomised controlled trial, Observational study design, Non-inferiority

## Abstract

**Background:**

Social inclusion is essential for an adequate rehabilitation process for people with serious mental illness (SMI). Various supported housing settings aim to promote housing competencies and social inclusion in service users. Nevertheless, there is a strong preference in service users for independent living. We aim to evaluate the effectiveness and efficiency of Independent Housing and Support (IHS) compared to institutionalised residential care settings and other treatment as usual conditions (RCS/TAU) in two cities in Switzerland.

**Methods:**

This is a prospective multi-centre, four-arm, non-inferiority cohort study investigating the effectiveness and efficiency of IHS and RCS/TAU for people with SMI. Effectiveness will be measured by a standardised measure of social inclusion as primary outcome as well as by measures of functioning and well-being. Efficiency will be analysed on the basis of service usage and costs associated with the different housing settings. Participants will be consecutively recruited and subsequently enrolled between April 2019 and December 2020 and assessed at baseline and after six, twelve and after 24 months. At one study site, 56 participants will be randomly assigned to one of the conditions; the other study site will be conducted as an observational study investigating 112 admitted participants.

**Discussion:**

While the UN Convention of the Rights of People with Disabilities aims to promote the opportunity to choose one’s place of residence, the limited supply of alternative forms of housing does not guarantee genuine freedom of choice. Increased diversification and flexibility of housing support is essential. If IHS shows non-inferiority in terms of their effectiveness and efficiency, users should be allowed to choose their kind of housing support.

**Trial registration:**

ClinicalTrials.gov: NCT03815604, December 04, 2019.

## Background

Along with the deinstitutionalization which has taken place since the 1960s, psychiatric care has made a shift from inpatient care to community based services [1]. As a consequence, housing and housing rehabilitation of people with serious mental illness (SMI) and their social inclusion became major elements of mental health care. However, housing rehabilitation for people with SMI is a neglected research field, and its implementation is largely based on the knowledge from the 1980s/90s [2].

With the UN Convention on the Rights of Persons with Disabilities [3], a basis has been built for people with disabilities to achieve a range of services according to their choice. Article 19 in particular aims to enable people with SMI a) to “have the opportunity to choose their place of residence”, b) to “have access to a range of in-home, residential and other community support services” and c) that services and facilities are “available on an equal basis to persons with disabilities and are responsive to their needs” with the goal of social participation and inclusion. Unfortunately, the implementation of the article has not advanced greatly since its passing, and allocation is more often driven by availability than by users’ choice or needs [4].

The common approach in psychiatric rehabilitation in many countries relies on a linear continuum of residential services that aims to enable the person with SMI to eventually live independently. This continuum contains several settings of different amounts of support and restrictiveness. In each of these settings, users are to become stabilised and learn specific housing skills. Once the client’s level of functioning improves, he or she “graduates” to move to a more independent setting [1]. However, current state of research shows clearly that most people in question do not reach the goal of living independently and remain in institutionalised residential care settings [5–7].

In line with the UN Convention, there is another form of residential rehabilitation called the Supported Housing model [1]. The model aims to place a person without prior housing arrangements directly into accommodation in the community, even if the individual is in need of specialized psychiatric or psychosocial care. The accommodation is independent of therapy or care and is rented by the service user according to his or her choice. Unlimited support is provided by off-site professionals concerning all aspects related to keeping the accommodation and according to individual needs. The main goal is seen in the social inclusion of service users into real life networks.

The heterogeneous terminology and conceptualization of various existing housing models complicate a comparison of different residential support services. Several attempts at creating a taxonomy were undertaken [8–10]. However, there is currently no consistent and widely acknowledged taxonomy available, and same setting labels often have different conceptualisations. The supported housing approach is sometimes entitled “supported” or “supportive” housing, whilst in England it is often called “floating outreach” or “floating support services”, e.g. [4, 11]. Based on the UN Convention on the Rights for Persons with Disabilities [3] and on early conceptual work in the US by the National Association of the State Mental Health Program Directors cited in Hogan and Carling [12] the supported housing model in this study is entitled Independent Housing and Support (IHS). The institutionalised residential rehabilitation services following the linear continuum of residential support services are labelled as residential care services and other treatment as usual conditions (RCS/TAU).

From a service user’s perspective, the supported housing approach has several advantages compared to the linear continuum approach of residential rehabilitation. Firstly, users of the supported housing model live in their own accommodation and not just in a housing setting. Experiencing one’s place of residence as one’s home is associated with more autonomy, empowerment and personal recovery [13–15]. Secondly, users do not have to move following a change of their needs. Due to the fluctuating nature of mental disorders and the requirement for a stable environment to maintain health, there is a recommendation for offering unlimited length of stay [16]. Thirdly, support in gaining housing skills is related to real-world requirements and not just to the respective settings environment. As a consequence, the main goal of supported housing is social inclusion rather than being supported according to the state of disability.

A substantial number of supported housing programs are designed with a focus on homeless people. Most prominent are the *Housing First* approach [17] and the HUD-VASH program (*Department of Housing and Urban Development – Veterans Affairs Supportive Housing Program* [18]). These programmes were initially developed in the United States and Canada and aim to support housing stability or permanent accommodation without the requirement of prior abstinence or therapy. Housing First programmes for homeless people are outperforming RCS in in many outcomes, such as healthcare utilization, reduction of substance use or improvement of mental health [19, 20].

In contrast, evidence for residential rehabilitation approaches for non-homeless people is less clear [16, 19–22]. Unlike the supported housing models for the homeless, IHS for non-homeless people with SMI mainly focus on psychosocial outcome variables like reduction of rehospitalisation rates and improving social functioning [19]. On the one hand, comparative analysis showed inconsistent results and no significant differences in outcomes between IHS and RCS could be found [20]. On the other hand, studies focusing on non-homeless people vary greatly in their methodological quality and none of them were conducted as randomised controlled trials. Nevertheless, there are some promising results of IHS for people with SMI.

Several studies show people with SMI to be more socially excluded than comparison groups [23, 24]. Concerning independent housing, some studies suggest that these conditions are socially isolating and foster experienced loneliness compared to living in a group home [14, 25], while the latter is assumed to promote a sense of community [26]. In contrast to this notion, other researchers believe there is a risk in re-establishing social relationships which rely on interactions with RCS-staff to overcome loneliness [14]. Another study highlights the importance of having a choice in when to socialize and when to be alone, and revaluate the “alone time” as an important aspect of the recovery process and as a specific advantage of IHS [27]. Furthermore, there are studies showing service users of IHS to be more socially included [13] and engaged in community [28].

There are only a small number of studies investigating the costs of residential rehabilitation programmes, especially with regard to non-homeless people. In general, varying welfare state conditions between countries make it difficult to compare costs of residential support programmes. Nevertheless, researchers report significant differences in costs in favour of IHS [29] as well as better cost-effectiveness [30, 31].

Furthermore, there is a strong preference of service users for independent living. According to a recent meta-analysis, 84% of service users expressed the desire to live independently when asked about their housing preferences [32]. This finding replicated previous results from the 1990s [33]. However, there is a difference in the preferred support service between service users and their care givers and families. While service users tend to prefer more independent permanent accommodation, staff and family members usually prefer the person to reside in more supported settings [34, 35]. However, having an opportunity to choose one’s place of residence is associated with greater subjective quality of life and adaptation to community living [36] and largely contributed to mental health recovery and wellbeing [27].

### Methodological considerations

Up to now, no randomised controlled trial (RCT) has been conducted with non-homeless populations to evaluate the efficacy of IHS. The aforementioned strong preferences minimise the likelihood of the realisation of RCTs investigating residential rehabilitation approaches for people with SMI. One factor is that the living situation of non-homeless people is usually already determined and the feasibility of a random allocation to a particular housing condition is, thus, questionable. Another factor is that patient preferences are known to have a tremendous effect on non-enrolment into an RCT or a higher risk of dropouts in the non-preferred condition [37–39]. Furthermore, a recent feasibility study failed in supporting large-scale randomised trials due to a high level of “gate keeping” by staff and strong preferences of service users for one of the two types of supported accommodation [4].

RCTs are widely acknowledged to be the “gold standard” of comparative research. However, alternatives are needed where RCTs are not feasible. It is now widely acknowledged that observational study designs (OSD) may provide important results that seem to be comparable with results of randomised controlled studies [40–42]. Moreover, a Cochrane review of 14 reviews covering 1583 studies did not find any significant differences in effect estimates of general healthcare outcomes between randomised controlled and observational studies [43]. While there is somewhat the worry that OSD generate overestimated effectivity due to self-selected referral resulting in a selection bias, other authors underline the possibility of underestimating intervention effects due to the naturalistic and therefore more heterogeneous “real-life” settings, e.g., [44]. Generally, OSD are able to provide external validity rather than internal validity.

## Methods

### Aims of the study

Based on these findings, we aim to investigate both 1) a care-related and 2) a methodological goal with the current multi-centre study to answer the following research questions:

1. What are the effectiveness and efficiency of IHS compared to RCS/TAU for non-homeless persons with severe mental illness after 6, 12, and 24 months?
2. Do the RCT and the OSD yield similar results?

The primary objective is to evaluate the effectiveness and efficiency of IHS and to directly compare it to traditional RCS/TAU for non-homeless people with severe mental illness. Effectiveness will be determined by social inclusion, proposed as a key outcome for individuals living with mental disorders and, in terms of research, a global priority measure [45]. Efficiency will be analysed based on the costs associated with the service utilization of the different housing settings. As the political goal is seen in allowing a freedom of choice in where to live and which support services to utilise [3] and because the differences in outcomes of previous studies between IHS and RCS for non-homeless populations were only small or inexistent [16, 19–22], we utilise a non-inferiority hypothesis in our study. We hypothesize that IHS yields no poorer social inclusion outcomes than traditional RCS/TAU.

One study centre is going to conduct an OSD with propensity score matching methods and the other center plans to conduct an RCT while learning from the results of the recently failed feasibility trial of IHS [4]. Thus, to our knowledge, the present study will include the first RCT investigating housing conditions for non-homeless people with SMI. The secondary objective of this study is to directly compare the outcomes of the RCT with the OSD. Here, we utilise the hypothesis that the OSD will provide similar results to the RCT. If the hypothesis is confirmed, observational studies investigating residential rehabilitation programmes for the non-homeless population could be considered as equivalent to the RCTs so far solely conducted with respect to homeless people.

In addition, we will conduct secondary analyses to investigate the moderation, mediation and procedural factors in IHS and RCS/TAU that influence outcomes.

### Study design

The present field-study is a prospective, multi-centre, four-arm, parallel-groups, cohort, non-inferiority open trial comparing Independent Housing and Support (IHS) with traditional residential care settings and other treatment as usual conditions (RCS/TAU) for non-homeless people with SMI in two cities in Switzerland (Bern and Zurich). To conduct the RCT, a specific time window is used in Zurich where IHS is going to be newly introduced. Access to IHS will be limited for study participants during recruitment to the study, so the realisation of an RCT will be possible due to a scarcity of IHS settings. Because IHS is already well-established in Bern, a comparative observational study with propensity score matching will be conducted there. Both, the RCT part and the OSD part of this study aim to prove the non-inferiority of IHS compared to RCS/TAU.

### Sample size

The appropriate sample size is determined by the non-inferiority approach and was calculated for the primary outcome measure ‘Social Functioning Scale’ [46, 47]. This scale has been used in a subsample of a randomised Housing First trial, from where the necessary data has been taken [48]. For calculating the appropriate sample size of the RCT study site, the application ‘Power and Sample Size’ [49] was used applying the following parameters: Power: 0.9; significance level: 0.025; non-inferiority margin: 15; difference between treatments (group means): 111.2–106.7; standard deviation: 12; and allocation ratio: 1:1. This procedure yielded a sample size of 28 people to be included in each arm.

The OSD sample size is based on the calculation for the RCT. Thus, 28 participants will be included in the treatment arm. For propensity scoring, the many-to-one matching method was chosen. Here, it is recommended to have a 2–3 times larger control group to facilitate matching [50, 51]. Accordingly, 56 to 84 participants will be included in the control group.

### Eligibility criteria

Eligible participants are composed of the target population of the investigated residential rehabilitation settings IHS and RCS/TAU. All included study participants have a primary mental disorder according to the International Classification of Diseases, 10th edition (ICD-10), are aged between 18 and 65 years, are able to communicate in the German language, to give informed consent, and to take their medication if indicated. They are also able to handle out of pocket expenses or have an established assistance of a custodian/guardian if indicated. The presence of any of the following criteria will lead to the exclusion of a potential participant: severe learning disabilities, intoxication, delirium, dementia, lack of capacity, an indication for hospital treatment due to acute symptomatology at admission and acute risk to self-harm or harm to others.

### Participant recruitment

Participants will be recruited between April 2019 and December 2020 within the conventional pathways to gain access to psychiatric residential rehabilitation. At the RCT study site, participants interested in IHS will be consecutively recruited by study collaborators. After agreeing to participate, a study collaborator will explain the purpose and procedures of the study. If the participant gives informed consent, she or he will be subjected to randomisation. For each participant, the result of randomisation is enclosed in an envelope based on a randomisation sheet, which has been calculated before study start for more than 56 consecutive cases. For this calculation, the statistical software R has been used with the procedure for block-randomisation described at [52]. The randomization sheet is saved in a formal study folder only accessible to researchers not involved in the allocation procedure so that no spontaneous modifications can be made during the study enrolment. Subsequently, the treatment condition to which they were randomly assigned will be arranged and the participant is then enrolled. For each measurement point at the RCT study site, participants will receive ten Swiss Francs. The goal of this financial compensation is to minimise potential dropouts in the control group, which have to be assumed due to the strong preference for IHS [32, 33] and the associated difficulties in the enrolment of an RCT [37, 38], especially in the field of supported housing [4].

At the OSD study site, participants will be consecutively recruited by residential rehabilitation staff or by social workers at the mental health hospital they are discharged from. After being admitted to the respective treatment condition, a study collaborator will ask them to give informed consent. Consenting participants will be subsequently enrolled into the observational study.

At both study sites, service users are considered to be intent-to-treat (ITT) after using their respective residential rehabilitation service for at least one month. Participants not considered to be ITT due to withdrawal before one month will be excluded from the study. If participants leave their residential rehabilitation setting after using it for at least one month, assessments with participants will continue in order to investigate the further progress of residential service users. Each dropout occurring during the recruitment phase will be replaced by a new subject in order to ensure an adequate number of participants in in each condition according to the power analysis. In case of dropout, all corresponding data on the contact sheet will be deleted and the person will never be contacted again. However, study data assessed before will be retained. Recruitment is complete when the intended number of participants is reached, after 21 months’ recruitment period at the latest. For the methodological objective, every participant at both study sites will be asked to consent for further use of data in encrypted form.

All assessed data will be entered in REDCap [53]. Participants will be coded and no personal data will be disclosed. The assessors of each study site will store personal data in password-protected files in order to contact them for conducting the assessments on a drive of the university.

### Interventions

The study intervention called Independent Housing and Support (IHS) is a psychosocial residential rehabilitation programme and means direct placement of people with SMI in accommodation in the community, i.e., without prior housing arrangements. This complex intervention follows the “First place – then train” paradigm which is well-known from Supported Employment within vocational rehabilitation. The accommodation is usually a flat rented at service user’s choice, and is financed at their expense. The service user will be supported by an off-site residential coach concerning all aspects related to finding and keeping his or her accommodation, including contacts with the landlord, social environment, relatives, and administration. The residential coach will not be in charge of any issue related to treatment or therapy, but will cooperate with the service user’s psychiatrist or psychiatric service. The intensity of residential coach support will be flexible and according to the service user’s needs. IHS is not transitional, but permanent and without time limitation and is independent of therapy or care. The main goal is the social inclusion of service users.

The control condition RCS/TAU consists of traditional residential care settings (RCS) such as staffed or unstaffed care homes, shared or non-shared apartments rented by the residential agency, and other treatment as usual conditions (e.g. host families, unsupervised living). RCS settings are part of a continuum of residential services other than hospital for non-acute support that aim to enable the individual with SMI to live independently. Users live in a group in a single or shared room, and receive daily support on-site or at least 7 h per day and at least on 5 days per week. If there is no continuous support on-site, users get 24 h on-call support. The hiring contract is concluded with the residential agency. In some of the RCS settings, there is the expectation that users will move on within two years. Closed residential settings or traditional housing settings not fulfilling the above criteria will be excluded from the present study.

As both study conditions are part of psychiatric rehabilitation not providing any acute psychiatric care, temporary inpatient or outpatient stays in a (mental) hospital are seen as common and will neither affect their utilisation of the rehabilitation setting nor their participation in the study.

### Outcome assessment and measures

Participants will be followed for two years after enrolment. Outcome assessments take place at baseline (T0), after 6 months (T1), 12 months (T2) and after 24 months (T3). Data will be collected by interview with participants as well as by self-report questionnaires answered by participants in a paper-pencil format. Assessments with participants will be conducted by trained assessors at the participants’ accommodation or another place of their choice. Model fidelity as well as the costs of RCS/TAU will be assessed by interview with heads of each of the utilised residential rehabilitation service.

Social inclusion will be assessed as the primary outcome as the main goal of residential rehabilitation approaches is the social inclusion of individuals with SMI. Secondary outcomes include functioning, quality of life, mental state, diagnoses, needs, capabilities, life events, social support, service utilization and costs. Participants’ diagnoses as well as contact frequency in IHS settings are collected from clinical records. All outcome measures will be assessed with a German version of the corresponding measures.

#### Social inclusion

The *Social Functioning Scale* (SFS) [46, 47] was developed specifically for adults with SMI and is used as the primary outcome measure to assess participants’ social inclusion. The 76-item questionnaire consists of seven subscales assessing participants’ social engagement/withdrawal, interpersonal behaviour, independence-performance, independence-competence, recreation, pro-social activities, and employment/occupation. Most of the items are to be answered on a 4-point Likert scale, and higher scores indicate better social inclusion. Raw total score range between 0 and 135 and each subscale score can be translated in scale scores with *m* = 100 and *SD* = 15. The German version showed good internal consistency and high validity and turned out to be comparable with the original English version [47].

#### Functioning

Participants’ functioning will be rated with the *Health of the Nation Outcome Scales* (HoNOS) [54] and with the modified scale of the *Global Assessment of Functioning Scale* (m-GAF) [55]. The HoNOS includes twelve items to be rated on a 5-point Likert scale. Total score range between 0 and 48, with lower score as indicator for better functioning. The German version of the rating scale (HoNOS-D) showed satisfactory feasibility, reliability and validity for most of the items [56, 57]. The m-GAF measures illness severity based on psychopathology and functioning on a 1 to 100 scale with higher score as indicator for better functioning. When possible, functioning will be rated by participants’ key workers, or by study collaborators when that is not possible (e.g. in some control condition situations or after the utilisation of IHS).

#### Mental state

For assessing mental state, the 9-item *Symptom Checklist* (SCL-K-9) [58] questionnaire will be used. Findings of its evaluation indicate the SCL-K-9 to be a suitable and efficient instrument for screening psychopathologic symptomatology with mostly satisfactory psychometric properties [59]. The questionnaire asks mental health problems on a 5-point Likert scale resulting in a total-score range from 0 to 36, with lower score implying better mental state.

#### Quality of life

The *Manchester Short Assessment of Quality of Life* (MANSA) [60] will be used to evaluate participants’ quality of life. This interview asks for participants’ satisfaction in general and with 16 different life domains in particular. Most items are to be rated on a 7-point Likert scale, with higher score indicating higher quality of life. The psychometric properties of the English version emerge satisfactory. The German version was translated by (Röpcke B, Linau N. Manchester Short Assessment of Quality of Life (MANSA) – Deutsche Fassung, unpublished).

#### Service utilisation

The evaluation of the German adaptation of the *Client Sociodemographic and Service Receipt Inventory* (CSSRI-EU) [61] proved its feasibility in assessing utilisation of mental health care in Germany. A study-specific adaptation to mental health care and residential rehabilitation settings in Switzerland (CSSRI-CH) will be used to measure costs and utilization of psychiatric services. The nine items ask participants for 1) their main source of income, 2) their net income in Swiss Francs, 3) their whereabouts in the last 12 months, 4) any health care utilisation for physical health problems and 5) for mental health problems, 6) any intake of psychotropic medication, 7) contact with criminal justice services, 8) internet access, and 9) any other support utilised. Evaluation will be conducted in a descriptive manner.

#### Support

For assessing emotional social support, the German adaptation of the *ENRICHD Social Support Inventory* (ESSI-D) [62, 63] will be utilised. The ESSI-D consists of five items to be answered on a 5-point Likert scale between “never” and “always” and proved to be an economic instrument with good reliability and satisfactory construct validity to assess emotional support in a sample of heart disease patients.

Additionally, 20 individual questions will be used to ask about important life events in the past six months, possibilities of self-determination, satisfaction with housing aspects, emotional and social support.

#### Needs

The *Camberwell Assessment of Need – Short Appraisal Schedule* (CANSAS) [64, 65] will be applied for assessing participants’ needs concerning 22 domains of health and functioning. The needs are ascertained by interview and rated on a 3-point scale (no problem; met need; unmet need). For interpretation, the met and unmet needs each will be summed-up. Inter-rater and test-retest reliability as well as validity showed to be reasonable to good [64], the psychometric properties of the German version were satisfactory [65].

#### Capabilities

The *Oxford Capabilities Questionnaire – Mental Health* (OxCAP-MH) [66, 67] is a 16-item questionnaire assessing participants’ capabilities on a 5-point Likert-scale. Total score ranges from 16 to 80 and can be translated into standardised score ranging from 0 to 100, with higher score indicating better capabilities. The scale shows good applicability as well as good validity and reliability [66, 67].

#### Service provision and costs

The mean number and duration of contacts with service users in IHS will be assessed with the Swiss medical tariff reimbursement tool (TARMED) that is currently the basis for outpatient reimbursement. This tool also allows to calculate the costs of service utilisation. Because TARMED only is used by the IHS settings, heads of each RCS/TAU service will be asked by a single item for the daily or weekly costs of their service during the fidelity assessments (see below).

#### Model Fidelity

The *Independent Housing and Support for people with mental disorders – Fidelity Scale* [Selbstbestimmtes Wohnen mit Unterstützung für Menschen mit psychischen Beeinträchtigungen – Modelltreue Skala (SeWo-Psych)] [68] will be used to assess model fidelity of IHS and to compare process quality of IHS and RCS/TAU programmes. This scale will be conducted as an interview with heads of the residential rehabilitation services (IHS and RCS/TAU) at the start of the recruitment phase at the respective service and one year after the last participant of the study has been recruited. The 32 items ask for the extent of agreement on the subscales 1) housing conditions, 2) team, 3) support conditions, and 4) orientation to inclusion in a 5-point Likert scale format. The scale has satisfactory psychometric properties. The SeWo-Psych refers to the *Quality Indicator for Rehabilitative Care* (QuIRC) [69] and is currently being validated in Germany.

The *Housing First Fidelity Scale* [70] will also be elevated. Housing First (HF) is a residential rehabilitation programme for homeless people in the United States and Canada [17]. The scale will be assessed at the same time points as the SeWo-Psych and will be utilised to enhance the comparability of IHS in Switzerland with the IHS of the Housing First program in North America. The 38-item interview asks heads of the residential rehabilitation settings to rate on a 5-point Likert scale the aspects of the subdimensions 1) housing choice and structure, 2) separation of housing and clinical services, 3) service philosophy, 4) service array, and 5) programme structure. Possible score range from 38 to 152 with higher score indicating higher fidelity with the HF program. The scale has shown promising utility, reliability, and validity [70].

## Data analyses

Relevant packages of the statistical software R will be used for conducting all statistical analyses [71]. For analysing and reporting, we follow the CONSORT guidelines for the reporting of non-inferiority and equivalence randomised trials [72, 73]. The main analyses will be performed using an a priori statistical analysis plan. For all analyses, Bonferroni corrections will be applied where necessary.

Missing data is expected due to refused items, missed appointments and drop-outs. With regard to recommended sensitivity analyses of missing data, we state the following assumptions about missing data: Missing data due to refused items is assumed to be independent of the respective treatment condition and to be completely related to the items refused. Thus, they are assumed to be missing at random (MAR). Due to the preference probably inherent in the investigated interventions and the associated difficulties [32, 37], missing data due to dropouts has to be assumed to be missing not at random (MNAR). Missing data will be analysed and imputation methods applied if appropriate [74, 75].

### Baseline characteristics

In the RCT sample, equivalence of baseline characteristics of the two study conditions IHS and RCS/TAU will be assessed descriptively as differences could have only occurred by chance.

To adjust for potential selection bias in the OSD sample, propensity score (PS) matching will be conducted. PS matching is a statistical method in which one case in the investigational condition is matched with one or more control cases based on the PS of each one, while the PS is the statistical probability of each case to receive the investigational condition based on relevant covariates [76, 77].

Relevant covariates for the PS model, meaning those covariates causally related to treatment condition and to the primary outcome of social inclusion, will be selected according to theoretical assumptions and empirical findings [78]. The propensity scores will be obtained by logistic regression [76, 79].

As matching method, many-to-one matching (m:1) with nearest neighbour approach (without replacement) will be applied [80]. In this method, every IHS participant will be matched with a variable amount between one to three participants of the RCS condition. This method is expected to generate the best matching quality with the lowest risk of bias [50, 51, 75]. Covariate balance will be checked before and after matching by recommended balance checks [50, 51, 77].

### Primary objective: care-related goal

For analysing the primary outcome variable social inclusion we will check for non-inferiority both in per-protocol and in imputed ITT samples to assess the security of inference of non-inferiority as recommended in the CONSORT guidelines for non-inferiority trials [72, 73].

Primary analysis will compare primary and secondary outcomes between IHS and RCS/TAU after 6, 12 and 24 months separately for both study sites. A repeated measure approach will be used where indicated. Non-inferiority of IHS compared to RCS/TAU will be accepted based on a 2.5% significance level if the lower bound of the 97.5% CI of M_IHS_-M_RCT/TAU_ is > δ = − 15, and vice versa, non-inferiority of IHS compared to RCS/TAU cannot be accepted if the lower bound of the 97.5% CI of M_IHS_-M_RCT/TAU_ is ≤ δ = − 15. This non-inferiority margin corresponds to one standard deviation of 15 scale scores (*m* = 100; *SD* = 15) on the primary outcome variable SFS [46, 47] and was chosen based on the clinical judgment of experts as no RCT investigating the SFS as outcome measure in a comparable sample was available [81]. In addition, assay sensitivity will be analysed by investigating pre-post differences in a first step. Assay sensitivity will be assumed, if there is a statistically and clinically significant pre-post increase in the SFS total scale score [82].

All other outcome measures will also be tested on their non-inferiority using the definition of margin δ as one standard deviation of the respective scale. Analyses will be extended to superiority testing in case of confirmed non-inferiority, and to interaction terms to explore mechanisms of treatment effects.

### Secondary objective: methodological goal

In order to analyse the secondary objective of methodology, descriptive homogeneity testing will be conducted to evaluate whether RCT data and OSD data can be pooled to be subsequently analysed jointly. If so, the analyses related to the care-related objective described above will be repeated with the pooled data from both study centres.

## Discussion

There is a strong preference in service users for independent living but a considerable lack of research into housing services in the context of mental health. In this prospective, four-arm cohort study we aim to evaluate the effectiveness and efficiency of Independent Housing and Support (IHS) compared to institutionalised residential care settings and other treatment as usual conditions (RCS/TAU) for people with SMI in two cities in Switzerland. Effectiveness will be assessed by social inclusion and related outcomes while efficiency will be analysed by service usage and costs testing hypotheses of non-inferiority. Participants will be enrolled between April 2019 and December 2020 and assessed at baseline, after six, twelve and after 24 months. At one study site, 56 participants will be randomly assigned to one of the conditions; the other study site will use an observational study design including 112 participants.

### Limitations

There are some limitations possibly compounding the interpretation of the findings. Firstly, most of the measures were assessed based on self-reported answers of participants, which can differ from more objective views. Therefore, the key workers ratings of participants’ functioning will be also assessed. Where necessary, key workers will be supported in doing the ratings by study collaborators. Secondly, blinding of participants or study collaborators is not possible due to the housing nature of the investigated conditions. Thirdly, the current study proposes a pragmatic non-inferiority hypothesis because there was no former study investigating our primary outcome variable *Social Functioning Scale* (SFS) [46, 47] in a comparable service field. Therefore, a historical justification of the non-inferiority margin δ and of an assay sensitivity as is usually recommended in non-inferiority trials [72, 73] was not applicable. Nevertheless, proposing a superiority hypothesis would not be appropriate for the present research because of the mixed results in comparative effectiveness research regarding residential rehabilitation in non-homeless people with SMI [16, 19–22]. Furthermore, the sample size calculation is based on the primary outcome measure, therefore, the present study has possibly not included enough participants to detect statistical significant differences in secondary outcomes tested on superiority of IHS. Accordingly, analyses on secondary outcomes have to be interpreted in an exploratory manner. It is important to recognise that the failure to find a difference does not imply non-inferiority regarding the respective outcome variable [83]. And last but not least, the study is conducted as a field study in two separate service areas. Therefore, the generalisability to other regions or other service providers may be limited.

### Challenges

In the RCT study part, the main challenge is seen in the risk of attrition bias due to the highly preferred IHS condition [32, 33, 37]. To minimise this risk, participants in the RCT study will receive ten Swiss Francs per interview and there will be a waiting list for all those interested in IHS refusing to be randomly assigned to the either condition. After the recruitment period finishes, participants on the waiting list will also be allowed to start with IHS.

In the OSD study part, the main challenge will be to reach the anticipated 84 participants in the control condition RCS/TAU in the foreseen timespan. This will be challenging for several reasons. Firstly, the strong preference of people with SMI for the IHS setting suggests that IHS users are more committed to the research project. Secondly, there are fewer admissions to RCS compared to IHS because there has to be a free bed available before someone can be admitted to an RCS. And thirdly, staff are assumed to be gate keeping, especially in RCS, as was the case in the study of Killaspy and team [4]. To overcome this challenge, more residential care facilities in the region are going to be recruited with the goal of reaching more admitting residents of RCS. Another possibility of coping with the sample size in the RCS/TAU condition is seen in applying the optimal matching method with propensity scores. Optimal matching is a matching-related method which does not strictly match individual participants but matches them with replacement to achieve overall minimal distance. This method allows for PS matching when there are not many appropriate control subjects to be matched with the subjects in the intervention condition [77].

## Data Availability

The datasets generated during the current study are available from the corresponding author after a written agreement with the principal investigators.

## Abbreviations

SMI: Serious Mental Illness
IHS: Independent Housing and Support
RCS/TAU: Residential Care Settings and other Treatments as Usual
RCT: Randomised Controlled Trial
OSD: Observational Study Design
ICD-10: International Classification of Diseases, 10th edition
CI: Confidence Interval
